# Design and Utility of Metal/Metal Oxide Nanoparticles Mediated by Thioether End-Functionalized Polymeric Ligands

**DOI:** 10.3390/polym8040156

**Published:** 2016-04-21

**Authors:** Shumaila Razzaque, Syed Zajif Hussain, Irshad Hussain, Bien Tan

**Affiliations:** 1 Hubei Key Laboratory of Material Chemistry and Service Failure, Key Laboratory for Large-Format Battery Materials and System, Ministry of Education, School of Chemistry and Chemical Engineering, Huazhong University of Science and Technology, Wuhan 437004, China; shumaila.chemist@gmail.com; 2 Department of Chemistry, Syed Babar Ali School of Science & Engineering (SBASSE), Lahore University of Management Sciences (LUMS), DHA, Lahore Cantt, Lahore 54792, Pakistan; zajifnano@gmail.com

**Keywords:** thioether end functionalized polymeric ligands, metal/metal oxide nanoparticles, fluorescent metal nanoclusters, drug delivery, magnetic resonance imaging (MRI), biosensing

## Abstract

The past few decades have witnessed significant advances in the development of functionalized metal/metal oxide nanoparticles including those of inorganic noble metals and magnetic materials stabilized by various polymeric ligands. Recent applications of such functionalized nanoparticles, including those in bio-imaging, sensing, catalysis, drug delivery, and other biomedical applications have triggered the need for their facile and reproducible preparation with a better control over their size, shape, and surface chemistry. In this perspective, the multidentate polymer ligands containing functional groups like thiol, thioether, and ester are important surface ligands for designing and synthesizing stable nanoparticles (NPs) of metals or their oxides with reproducibility and high yield. These ligands have offered an unprecedented control over the particle size of both nanoparticles and nanoclusters with enhanced colloidal stability, having tunable solubility in aqueous and organic media, and tunable optical, magnetic, and fluorescent properties. This review summarizes the synthetic methodologies and stability of nanoparticles and fluorescent nanoclusters of metals (Au, Ag, Cu, Pt, and other transition metal oxides) prepared by using thioether based ligands and highlights their applications in bio-imaging, sensing, drug delivery, magnetic resonance imaging (MRI), and catalysis. The future applications of fluorescent metal NPs like thermal gradient optical imaging, single molecule optoelectronics, sensors, and optical components of the detector are also envisaged.

## 1. Introduction

Metal nanoparticles have attracted a considerable interest because of their unusual/unique optical, electrical, magnetic, physical and chemical properties, distinctly different from those of their bulk analogues [1]. The size and shape of metal particles, especially when below 100 nm, has a profound impact on their physical and chemical properties [2,3]. These metal/metal oxide nanoparticles have been demonstrated to be the potential candidates for a number of biological and biomedical applications including *in vitro* diagnostics and *in vivo* imaging [4].

Bulk metals are well known to display electrical conductivity and pronounced optical reflection due to the free movement of electrons in the conduction band [5]. On the contrary, the metal nanoparticles (MNPs), especially those of gold, silver, and copper, exhibit a variety of colors, due to their characteristic Surface Plasmon resonance (SPR), depending on their size, shape, surface chemistry, and dispersion media. Metal nanoparticles are also gaining much attention because of their fluorescent nature, as the particle size approaches *ca.* 1 nm, and their band structure becomes discontinuous and is further broken down to discrete energy levels. These molecule-like metal nanoclusters (MNCs) are composed of a few to tens of atoms and have a size comparable to the Fermi wavelength of electrons, and do not possess any plasmonic behavior but display interesting fluorescent properties because of the charge transfer from the surface bound ligands to the metal core. Therefore, by tuning the size and, number of atoms in MNCs, the gap between discrete energy levels can be switched, offering a significant control over their absorption and emission wavelengths. On the basis of size dependent properties, these MNCS are the desirable candidates for applications in bio-imaging, sensing, optoelectronics, and catalysis, *etc.* [6].

In addition to the interesting optical properties of metal nanoparticles/nanoclusters and their subsequent applications, the magnetic nanoparticles like those of Fe_3_O_4_ and Co have also gained scientific interest for their unique magnetic properties such as superparamagnetism, high coercivity, low Curie temperature, and high magnetic susceptibility. Among these, the magnetic iron oxide nanoparticles (MIONs) have attracted enormous attention, owing to their low toxicity and high biocompatibility. They are considered to be among the most favorable candidates for bio-applications including those in magnetic resonance imaging (MRI), magnetic fluid hyperthermia (MFH), magnetic separation and immobilization of biomolecules such as nucleic acids and proteins, development of drug delivery systems for controlled release of drugs, bio-labeling, and magnetic sensors [7].

## 2. Synthesis and Assembly of Metal/Metal Oxide Nanoparticles

Numerous methodologies have been adopted for the synthesis of metal/metal oxide nanoparticles, which have their own unique set of advantages or disadvantages. The physicochemical properties of NPs can be tuned by proper control of their size and shape and by carefully selecting suitable surface functionalities. The selection of the proper synthetic method is thus pivotal in optimizing the size and shape of the nanoparticles and thereby tuning their properties for a given specific application. In this perspective, a general overview of various methods of preparation and the applications of these NPs, especially those involving the use of thioether end-functionalized polymeric ligands, is presented in this review.

Among the conventional methods of synthesis, the chemical reduction of metal precursors by using various reducing agents in the presence of appropriate stabilizers is the most common strategy especially when they are desired for biomedical applications. Turkevitch, in 1951, reported the synthesis of Au and Ag nanoparticles by the reduction of Au and Ag salts by citrate [8] in aqueous media. The synthetic protocol was further improved by Frens in 1973 and spherical AuNPs of different sizes (between 16 and 147 nm) were produced by adjusting the precursor/stabilizer ratios [9]. Since then, this approach has become very popular and a standard method for the synthesis of metal nanoparticles synthesis, especially for gold and silver, in aqueous media and several reports are now available demonstrating the mechanism and kinetics of these synthetic strategies [10]. A practical insight into this methodology has been achieved by the successful preparation of AuNPs by citrate reduction with well-defined size distribution (9 to 120 nm) [11].

In addition to the Frens–Turkevich method of preparing gold hydrosols, the Brust–Schiffrin method is one of the most leading strategies to obtain small metal NPs in organic media. In this method, nanoparticles are protected by organic thiol molecules to provide an effective control over the nucleation and growth of NPs. This method involves a simple protocol based on the fast chemical reduction of metal salts by borohydride at ambient conditions and capping the NPs at initial stages of their growth using thiol-based stabilizing ligands to obtain small sized NPs. The chemical reaction presented below shows the possible mechanism [12].

AuCl_4(aqueous)_ + N(C_8_H_17_)_4(toulene)_ → N(C_8_H_17_)_4_4AuCl_4(toulene)_ (1)

(2) $$\mathrm{mAu}{{{\mathrm{Cl}}_{4}}^{-}}_{\left(\mathrm{toulene}\right)}+{\mathrm{Cl}}_{12}H_{22}{\mathrm{SH}}_{\left(\mathrm{toulene}\right)}\overset{\mathrm{NaB}H_{4}}{\to }4m{{\mathrm{Cl}}^{-}}_{\left(\mathrm{aqueous}\right)}+{\left(\mathrm{Au}\right)}_{m}{\left(C_{12}H_{22}\mathrm{SH}\right)}_{n(\mathrm{toulene})}$$

This method succeeded over many other alternatives and has become the most widely employed procedure for synthesizing metal NPs in the *ca*. 1 to 8 nm range, using either a two-phase liquid/liquid system or a suitable single-phase system [12]. The Brust, *et al.* method has been subjected to different types of modifications for the preparation of several metal and semiconductor NPs, using different protecting ligands and reducing agents to obtain different sizes of metal nanoparticles such as Au, Ag, Pt, and Cu [13]. Therefore, extensive studies have been carried out to obtain NPs of desirable size by simple variations in the above mentioned procedures but practical constraints are apparent when monodispersity is required.

In this perspective, continuous efforts in the development of NP systems led to the selection of appropriate ligands crucial for tuning the morphology and size of metal NPs as well as controlling their long term stability in the biological and chemical environment. Therefore, depending upon their applications, the design and synthesis of customized metal NPs capped with a suitable ligand have always been desired. Polymers are among the promising motifs as stabilizers for specific NP chemistries and/or architectures and bestow desired surface properties to a given material without significantly altering other desirable properties of the material [14].

**Figure 1:**
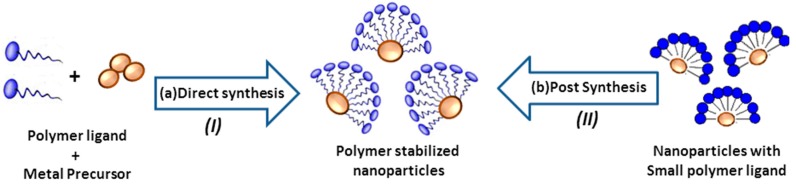
Schematic illustration of (**a**) direct synthesis (in situ) of nanoparticles (NPs) (**b**) Post synthesis capping by ligand exchange method for the polymer stabilized NPs.

Generally, the standard methodologies followed for the synthesis of polymer-ligated NPs fall into two general categories (Figure 1) [15].

- The NPs are formed in the presence of the polymer ligands (*in situ* formation)
- The small-molecular ligands are exchanged with polymer ligands on pre-formed NPs (ligand exchange)

Polymeric ligands provide several possible advantages over small-molecule ligands [16] although small molecules provide a certain degree of physical barrier that results in a smaller hydrodynamic radius that favors the *in vivo* applications for efficient trans membrane permeation and excretion [17]. However, the shell thickness of ligands around NPs is too thin; it reduces their steric barrier and further leads to their aggregation by enervating their stability. Polymeric ligands, therefore, play a vital role in nanoparticles synthesis as they surround the NPs with a substantial physical barrier preventing the core NPs from coming into contact before they are collapsed. As a consequence of core separation, however, there is an increase in the hydrodynamic radius of the nanoparticles, a phenomenon generally known as steric stabilization [18].

Multiple repeating units exist in the polymer backbone, each of which is capable of displaying different functionalities of the ligand. Such polymers possess a greater potential to stabilize metal nanoparticles due to their multi-valency. The co-polymers are also useful to introduce frequent incorporation of multiple types of ligand functionalities [19]. On the other hand, small molecular ligands typically require very strong affinities for the surface of the nanoparticle to ensure their stability. Thus, multivalency of polymeric ligands enhances the binding effect of the ligating species (pyridines, *N*-alkyl pyrrolidones or thioethers) with metal nanoparticles, which cannot strongly bind with metal NPs [20]. The accessibility to the nanoparticle surface and thickness of the stabilizing layer are dictated by the size as well as the ratio of polymeric ligand to metallic precursor [21,22]. The utilization of capping ligands is not only associated in terms of stability but also in providing suitable synthetic conditions and the ability to tune the size and shape of the nanoparticles. In addition to the above features, the ligands have a strong impact on sensing, imaging, optoelectronic, and magnetic properties of metal NPs due to the differences in their effect on the electronic and binding properties. For this purpose, polyethylene glycol (PEG), carbohydrates and polymeric ligating species, a number of alkyl thiols and sulfur containing moieties have been demonstrated for stabilization of metal nanoparticles [23]. Alkyl thiols are among the best capping agents for stabilization of metal NPs, where thiol molecules covalently bind to metal forming a strong thiol-metal bond and are particularly the best complimentary ligands for metals like Au, Ag, Pd, and Pt. A wide variety of different thiolated molecules can be placed in the ligand shell, which impart an extra colloidal stability to NPs [24,25]. Au and Ag, in particular, show strong affinity for thiols (e.g., monolayer-capping via two-phase synthesis, place-exchanging, stepwise assembling, DNA linking, or hydrogen bonding) [26]. Other than alkyl thiols, many other sulfur containing functional moieties like xanthates and thiocarbamates can also be used but they have less affinity towards metals as compared to alkane thiols [27].

**Scheme 1:**
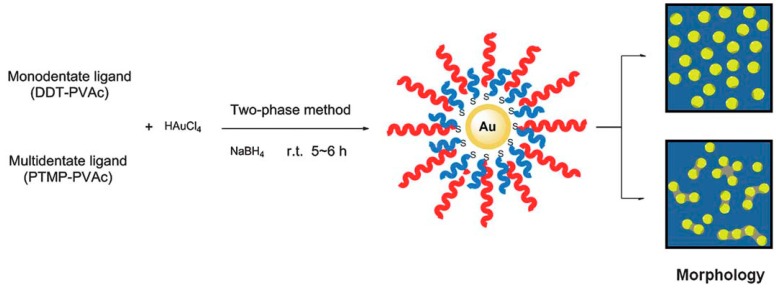
Schematic illustration for the preparation of Au nanoparticles (NPs) stabilized by thioether polymer ligands [29].

In addition to the nanoparticles synthesis, numerous methodologies for their self-assembly have also been successfully established to have a better control over the inter-particle distance and spatial properties by addressing the molecular-level understanding of inter-particle interactions and reactivities [28]. The development of polydentate ligands has resulted in numerous applications in the field of nanoparticles self-assembly. Specifically, hydrophobic and hydrophilic end-functionalized polymers [29] have been designed and used to produce NPs through the conventional Brust-Schiffrin method (Scheme 1). The use of polythioethers, containing highly polarizable donor group of atoms, e.g., sulfur atom of thioethers, which confer access to the metal NPs [26], has particularly been exploited in this regard. The exploration of thioether based coordination chemistry has led to the design of molecular assemblies with unique properties having tailored framework dimensions while providing chemical reversibility. Thioethers mimic the phosphine ligands, being soft electron donors, but are comparatively less toxic and easy to handle in air [30]. They can act as adsorbate molecules with multiple attachment points to create well ordered, functional monolayers on metal NPs via thioether functionality. As compared to thiols, thioethers result in a less strong metal-S interaction, but the combination of multiple thioether-metal interactions can lead to stable and well-ordered monolayers on the metal surfaces [31].

### Synthesis of Thioether Polymer Ligands

**Scheme 2:**
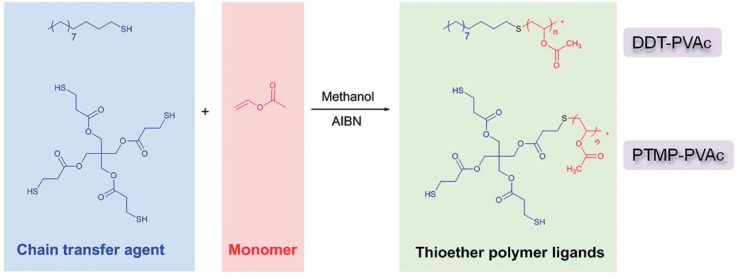
Schematic illustration of thioether polymer ligand formation [29].

As far as the control over size and monodispersity of NPs is concerned, the thioether ligands are now among the attractive polymer stabilizers to produce uniform metal NPs in aqueous/organic medium thus offering a very useful system to control the size distributions and facile functionalization of nanoparticles for a variety of applications. In this regard, our group has been actively engaged and has reported decent strategies for the facile preparation of a hydrophilic and hydrophobic thioether end-functionalized polymer library, and demonstrated their use to produce a variety of metal/metal oxides. For example, we have reported the synthesis of thioether end-functionalized polymer ligands, following the typical chain transfer methodology, using thiols (or diol/multi-thiols) as the chain-transfer agent (Scheme 2). An important feature of this methodology is that it leads to low molar mass oligomeric species with relatively narrow molecular weight distributions (PDI < 1.5). The unreacted free thiol can be easily removed by polymer re-precipitation in a solvent which is considered to be a good solvent for the thiol chain transfer agent.

The Brust–Schiffrin method is associated with the issues related to the difficulty in replacing strong interacting thiol molecules on their surface with other ligands leading to a slow ligand exchange that mainly results in a mixture of ligands at the surface of nanoparticles [29]. The thioether end functionalized polymer ligands, however, have relatively weaker interaction with the metal surfaces, thus rendering metal NPs more prone to post-synthesis functionalization by relatively easier and rapid ligand exchange to get a better control over the nanoparticles’ surface chemistry.

## 3. Preparation of NPs Using Water Soluble Thioether End Functionalized Polymeric Ligands

Although the atomic composition of nanoparticles is more important in determining their physical properties, the chemical nature of the capping ligands dictates their wettability by solvents. For desirable biological applications, the stabilizing polymeric unit ensures water solubility and long term stability toward the surrounding environment [18]. There are many promising polymeric ligands, which can improve the NPs water solubility, biocompatibility and system circulation time [32]. The most commonly adopted ligands are, however, usually based on poly(ethylene glycol) (PEG) [33] and carbohydrates such as starch, dextran [34], and chitosan [35]. PEG is an appropriate ligand for NPs capping as it fulfills the requirement of long circulation times in the blood, reduces the degree of opsonization, and provides excellent long-term stability in high salt concentrations and extreme pH conditions [36]. Such molecular species are suitable for dispersing NPs in aqueous media for biological applications and also allow their electrostatic stabilization [37]. For instance, the commonly used small molecule of PEG is *ω*-thiol functionalized alkane ether of tetra(ethylene glycol), which is used to stabilize Au NPs for aqueous systems [38]. In a similar manner, the ethylene glycol chain can also be modified to provide chemical functionality that leads to electrostatic stabilization [39]. The use of disulfides, thioethers, or tetradentate thioether ligands as capping agents is also well explored to mediate the formation of spherical or related assemblies of metal NPs. Such ligands precisely control the morphology of metal NPs and add remarkable functionalities to exploit their biomedical applications such as sensing, bio-imaging, and drug delivery [28].

**Figure 2:**
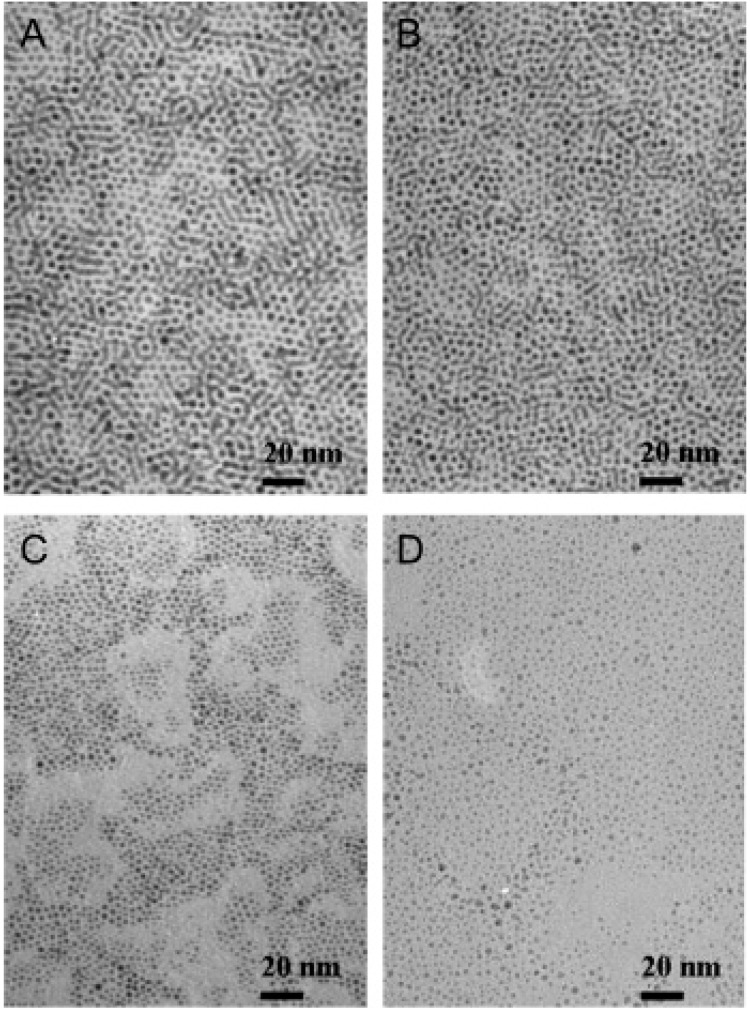
Transmission electron microscopy (TEM) micrographs of Gold NPs with various concentrations of polymer ligands. (**A**) 0.006mM (**B**) 0.03mM (**C**) 0.6mM and (**D**) 3.6mM [41].

The novelty of a thioether as a capping agent was firstly explored by Reinhoudt, *et al.* in 2001. Different derivatives of thioethers were prepared by varying the chain length as well as the number of thioether functionalities and used as a passivating agent to prepare self-assembled monolayers on the surface of gold nanoparticles following the Brust method [40]. An elegant strategy to control the size of gold nanoparticles in the range of 1–4 nm (Figure 2) was demonstrated by Hussain, *et al.* They used poly methacrylic acid (PMAA) functionalized with alkyl thioether as stabilizer and by using various ratios of polymer to gold, were able produce water soluble and fairly monodisperse Au NPs in the size range of 1–4 nm [41]. Encouraged by this work, Tan further investigated the effect of water soluble polymeric ligands with different monomers as a repeating unit in a polymer backbone used as a stabilizing agent to produce the water soluble AuNP [42].

### 3.1. Characteristic Properties of NPs Capped with Multifunctional Water Soluble Polymeric Ligands (MWP)

Water-soluble polymers which are primarily used as capping ligands for the metal or metal oxide NPs include but are not limited to: polyethylene glycol (PEG) [43,44,45], polyvinyl alcohol (PVA) [46,47,48], polyvinyl pyrrolidone (PVP) [49,50,51], dendritic polyamido amine (PAMAM) [52], polyethyleneimine (PEI) [53,54,55], mercaptan and acrylic polymers such as pentaerythritol tetrakis (3-mercaptopropionate) polymethacrylic acid (PTTM–PMAA) [56,57] and dodecanethiol polymethacrylic acid (DDT–PMAA) [41]. Some natural polymers such as starch, dextran, chitosan, plant resins, and other bio-macromolecules like polynucleic acids and polypeptides can also be employed as capping agents [58,59,60,61]. Modification of the NPs using these multifunctional polymeric ligands as capping agents mainly has the following advantages.

### 3.2. Control Over Particle Size and Morphology

Polymers are promising candidates for the stabilization of nanoparticles during the course of synthesis either by serving as scaffold for immobilizing the NPs or by interacting with the nanoparticles’ surface covalently/electrostatically, and preventing them from aggregation, and also by modulating the spatial distribution of the nanoparticles. The presence of abundant functional groups on the polymer back bone offers better affinity/interaction with the surface atoms of growing NPs and can prevent them from further growth into larger particles/aggregates [15]. Many of the polymers also play an essential role in tuning the size, shape, and organization of NPs in addition to stabilization. The size, monodispersity, and morphology of NPs can be fairly precisely controlled by adjusting the concentration and molecular weight of the polymeric ligands to the metal precursors [42,57,62]. The precise control over the size and shape of Au NP assemblies has been well explored by the use of multidentate thioether ligands. The stable spherical assemblies of Au NPs in the size regime of 30–80 nm have been prepared by using multidentate ligands such as tetra-[(methylthio)methyl]silane Si(CH_2_SCH_3_)_4_ (TTE), and methyltris[(methylthio)methyl]silane, MeSi(CH_2_SCH_3_)_3_). Similar nanoparticle assemblies prepared by using thiolated ligands can be readily disassembled [26]. More pronounced control over the size and spherical morphology was attained by utilizing the mediator-template protocol, which showed that the spherical assembly of Au NPs is dependent upon the geometric ratio of surfactant molecules and the number of thioether functionalities. Thus, the combination of ligand meditation, the surface templating, and their relative concentrations serves as a driving force to establish the interparticle linkages and stability. The key factors for the strong interparticle interactions and stability, is the molecular driving force exerted by the mediator molecules like thioethers and hydrophobic surfactants, acting as templates as well as capping agents [31]. Obare and coworkers also demonstrated a novel method of synthesizing Pd nanoparticles by using thioethers as capping agent [30]. Compared with the conventional approaches, these NPs featured significantly uniform morphology, stability, and monodispersity with particle size in the range of 1–3 nm. This control over particle size and distribution was tuned by modulating the reaction time and temperature.

**Figure 3:**
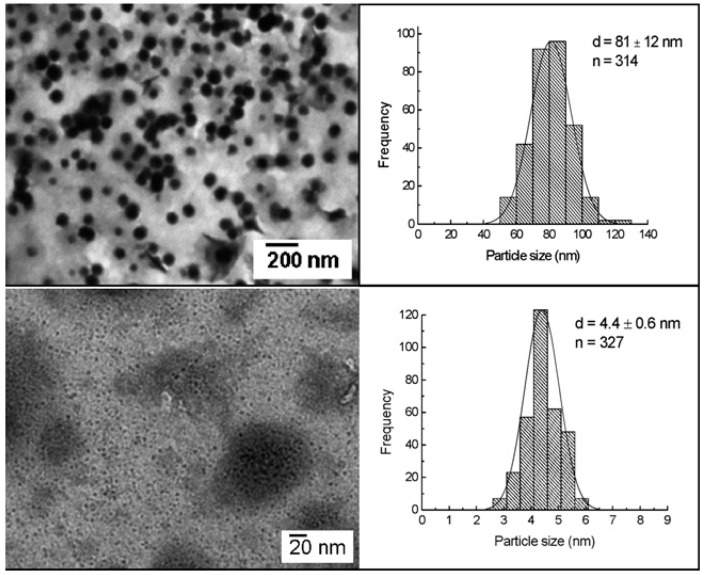
TEM micrographs and the corresponding size distribution histograms of the Co NPs synthesized by rapid injection (**top** panels) or drop-wise addition (**bottom** panels) of the reductant NaBH_4_ into a mixture of CoCl_2_ solution and the polymer [62].

Lu, *et al.* demonstrated a one-step protocol for monodisperse, water-soluble Co NPs with tunable size and shape through a wet chemical reduction method using alkyl thioether end-functionalized polymer ligand poly(methacrylic acid) DDT-PMAA as capping ligand [62]. Through the optimization of molecular weight and concentration of the DDT-PMAA, they were able to obtain ultra-small monodisperse spherical nanoparticles (2–7.5 nm) as well as larger spherical Co NPs (80 nm) (Figure 3). The aqueous solution of Co NPs was highly stable for several weeks and showed super-paramagnetism at room temperature. Due to the presence of a large number of carboxylic acid (COOH) groups on their surface, these NPs were demonstrated to be effective for numerous bio-applications through easier post-synthesis chemical functionalization.

### 3.3. Stability of Nanoparticles

Colloidal stability of NPs is based on inter-particle behavior resulting from intermolecular and surface forces (e.g., van der Waal’s forces, the repulsive electrostatic double layer (EDL), and structural forces such as depletion attraction). The overall balance among these forces determines the colloidal stability of NPs in an aqueous suspension [63]. NPs tend to aggregate because of their high surface area as well as large surface energy [64]. Nanoparticles aggregation phenomenon is more pronounced in biological medium because of interaction with bio-macromolecules. Therefore, a variety of approaches have been demonstrated to avoid the formation of aggregates including: electrostatic, steric, or electrosteric stabilization [65].

The utilization of MWPs as capping agent can prompt the mechanisms of electrostatic repulsion, steric hindrance, and vacancy stabilization effects, by changing the physical and chemical properties of the NPs surface, to keep them stable for a longer period of time [66]. In many cases MWPs also provide excellent stability against chemical oxidation of NPs in aqueous medium which was otherwise not possible. For instance, Co NPs possess a highly oxidative nature, but when coated with DDT–PMAA, they showed better stability in air for several days [62]. Likewise, DDT–PMAA protected MIONs showed outstanding stability towards oxidation and aggregation [7].

### 3.4. Post-Synthesis Functionalization of Nanoparticles

The multidentate thioether ligands have multiple functional groups that lead to an ease in post-synthesis NPs functionalization to better control their surface chemical properties. MWPs usually possess a variety of functional groups in their backbone [67]. The post-synthesis functionalization may be in terms of host-guest interactions or direct chemical modification, which yield NPs with multifunctional properties that are useful to further broaden their scope of applications. Host–guest interactions refer to the process where two or more chemical functionalities combine through weak electrostatic interactions or hydrogen bonding. Thus the functional groups present at the NPs surface act as host molecules and can interact with a variety of guest molecules selectively to form a supra-molecular system required for a particular application [68]. For instance, the hexane solution of monodispersed ferric oxide NPs or silver NPs stabilized with oleic acid and dispersed in hexane can interact α-CD to facilitate their phase transfer from the organic to the aqueous phase because of complexation between α-CD with oleic acid [69].

The presence of functional groups like carboxyl, amino, and hydroxyl group *etc.* in a polymer backbone coated on the NPs surface can be chemically modified through characteristic chemical reactions such as esterification, acylation, condensation *etc.*, which lead to a change in the chemical properties of the NPs. Latham *et al.* used trifluoroethylester-polyethyleneglycol-thiol polymer (TFEE–PEG–SH) as capping ligand to prepare the water-soluble gold and FePt alloy magnetic NPs [70]. TFEE-PEG-SH is highly soluble in water while the trifluoro ether functionality is a good leaving group, aiding in the easier replacement with other chemical moieties for further functionalization. It is, therefore, inferred that the presence of different functional groups in polymers offers an easier control over the surface chemistry of NPs for various applications, in addition to their role to control NPs’ size and stability.

## 4. Organic Medium Soluble NPs

The polymer ligands are usually attached to the NPs surface either through single or a combination of a variety of different interaction modes. These interactions include electrostatic forces, hydrophobic/hydrophilic interactions or covalent bonding between surface atoms of NPs and the functional groups present on the polymer back bone. The ligand exchange on the NPs’ surface using the difference of interaction between ligands and NPs can aid the transfer from organic to aqueous phase and make them suitable for a particular bio-application. Traditionally, the phase transfer (PT) of nanoparticles has been accomplished by utilizing tetra-alkylammonium salts such as tetra-octylammonium bromide (TOAB) for the phase transfer of Au NPs; 4-(dimethylamino)pyridine (DMAP) for Au and Pd NPs, hexadecyltrimethylammonium bromide (CTAB) [71,72] for magnetic NPs (MNPs), and other amphiphilic species such as 2,3-dimercaptosuccinic acid (DMSA) [73], cyclodextrin [69], and copolymers [74] for the phase transfer of oleic acid capped NPs. Another frequently applied methodology is the ligand exchange in which the NPs are customized according to their desired applications; which utilizes water soluble polymeric ligands. In this technique, the ionic stabilizing agents of previously prepared metal nanoparticles are replaced with new ones, e.g., alkane thiols and their derivatives are used to replace the existing ionic stabilizing agents like citrate or tetra-octylammonium bromide (TOAB) [75]. The higher stability of thioether stabilized gold colloids in organic solvents is generally attributed to the arrangement of these passivating ligands in a well ordered monolayer fashion in addition to the multiple thioether-Au interactions [40]. Similarly, Maye *et al.* prepared hydrophobic Au NPs through a mediator template route to control the size of NPs assemblies via maintaining the relative ratios of tridentate thioether as a mediator and TOAB as a templating agent [31].

**Figure 4:**
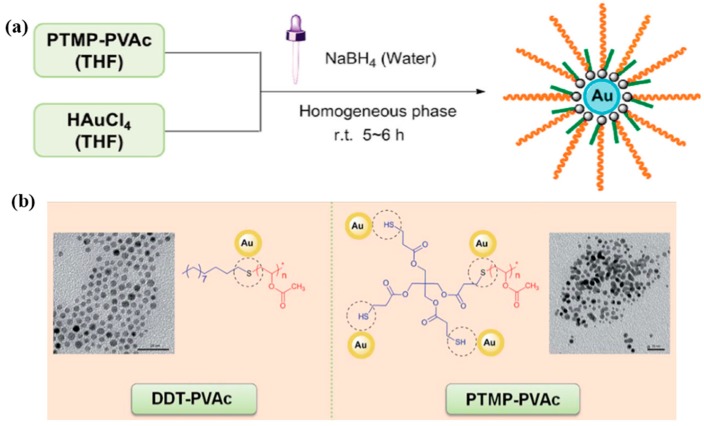
Scheme illustrating (**a**) The preparation of AuNCs in organic solvent; (**b**) TEM images of AuNCs capped by thioether end-functionalized ligands. The scale bar is 20 nm.

Recently Tan, *et al.* demonstrated a very simple protocol for the synthesis of PTMP–PVAc protected Au nanoclusters (NCs) without employing any phase transfer reagent. These NCs showed a good solubility in a variety of organic solvents as well as high stability for several months. Ag and Pt NCs have also been prepared following the same method [6]. Moreover, the impact of ligands on the Au NCs morphology was also studied, which showed that the multidentate (PTMP–PVAc) ligand capped NCs had a special “multimer” morphology in organic phase while monodentate thioether end functionalized ligands (DDT–PVAc) produced fairly uniform NCs (Figure 4). The hydrolysis of such ligands improves the hydrophilic property that results in phase-transfer from organic to aqueous media. Thus it is a simple and an easy approach to functionalize Au CPs that could possess a variety of applications including optoelectronics, catalysis, and sensing [29].

## 5. Magnetic Nanoparticles

Magnetic nanostructure materials have numerous potential applications in the field of ferrofluids, catalysis, information storage, as well as in biomedical applications such as magnetic resonance imaging (MRI) [76,77], magnetic fluid hyperthermia (MFH) [78], magnetic separation of drug molecules, and drug delivery, *etc.* The fabrication of practically useful magnetic nanostructures with enhanced stability and high susceptible magnetization is crucial for a number of bio-applications [79]. Numerous methodologies are, therefore, being explored/adopted for the reproducible preparation of magnetic iron oxide nanoparticles (MIONs) such as micro-emulsion processing, ultrasound and photo-irradiation, polyol synthesis [80], thermal decomposition of metal organometallic compounds [81], laser pyrolysis, hydrothermal, and the most commonly applied co-precipitation route [82,83]. In the aqueous precipitation approach, water soluble polymer ligands can produce NPs with efficient control over their size, shape, mono-dispersity, morphology, excellent biocompatibility, and good environment adaptability due to the presence of long chains bearing the variety of functional groups like OH, NH_2_, SH, and COOH. Thus, magnetic NPs with small size and better water solubility are desirable candidates for many biological systems [67,84].

**Figure 5:**
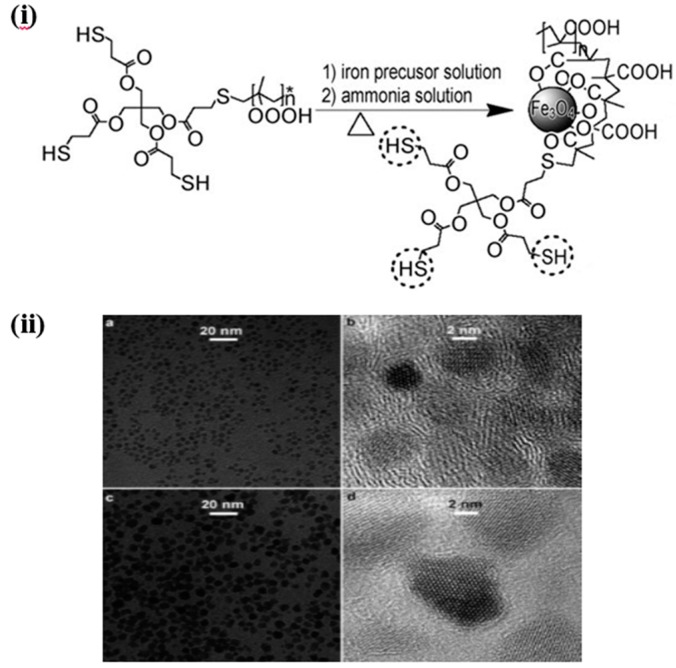
Scheme illustrating the (**i**) Synthesis of MIONs; (**ii**) TEM images (**a,b**) 4.5-nm and (**c,d**) 8.5-nm PMAA–PTTM protected Fe_3_O_4_ nanocrystals. The molar ratio between carboxylic acid groups and FeCl_3_·6H_2_O in both cases is 3:4 [57].

In 2008, Li, *et al.* reported the synthesis of water-soluble magnetic iron oxide NPs (MIONs, *D* = 4.5 ± 0.4 nm) using a multifunctional water-soluble polymer ligand (PTTM–PMAA) through a co-precipitation method. (Figure 5) It was found that the ratio between carboxylic acid groups present in the polymer ligand and iron precursors played an important role in determining the particle size distribution of the MIONs. The free thiol (–SH) and carboxylic acid (–COOH) functional groups of the polymer ligand were exploited and post-synthetic modification of these NPs led to water-soluble fluorescent magnetic NPs through coupling with fluorescent dyes. Further improvement was made by selecting a high-temperature co-precipitation method to prepare ultra-small magnetic iron oxide nanoparticles (UMIONs, 3.3 ± 0.5 nm) using thiol-functionalized poly(methacrylic acid) (PMAA–PTTM, *M*_n_ = 6360 g·mol^−1^, *M*_w_ = 7520 g·mol^−1^) as a stabilizer, which shows better control over the particle size and size distribution compared with other polymers used for the same purpose. These polymer ligands can effectively prevent the growth and aggregation of nanoparticles to ensure smaller size of the nanoparticles. The carboxylic functional group also aided in the water solubility and stability of NPs over a wide range of pH.

**Figure 6:**
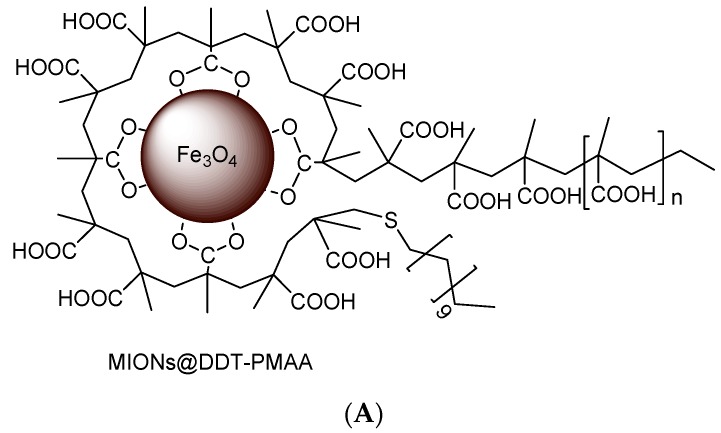

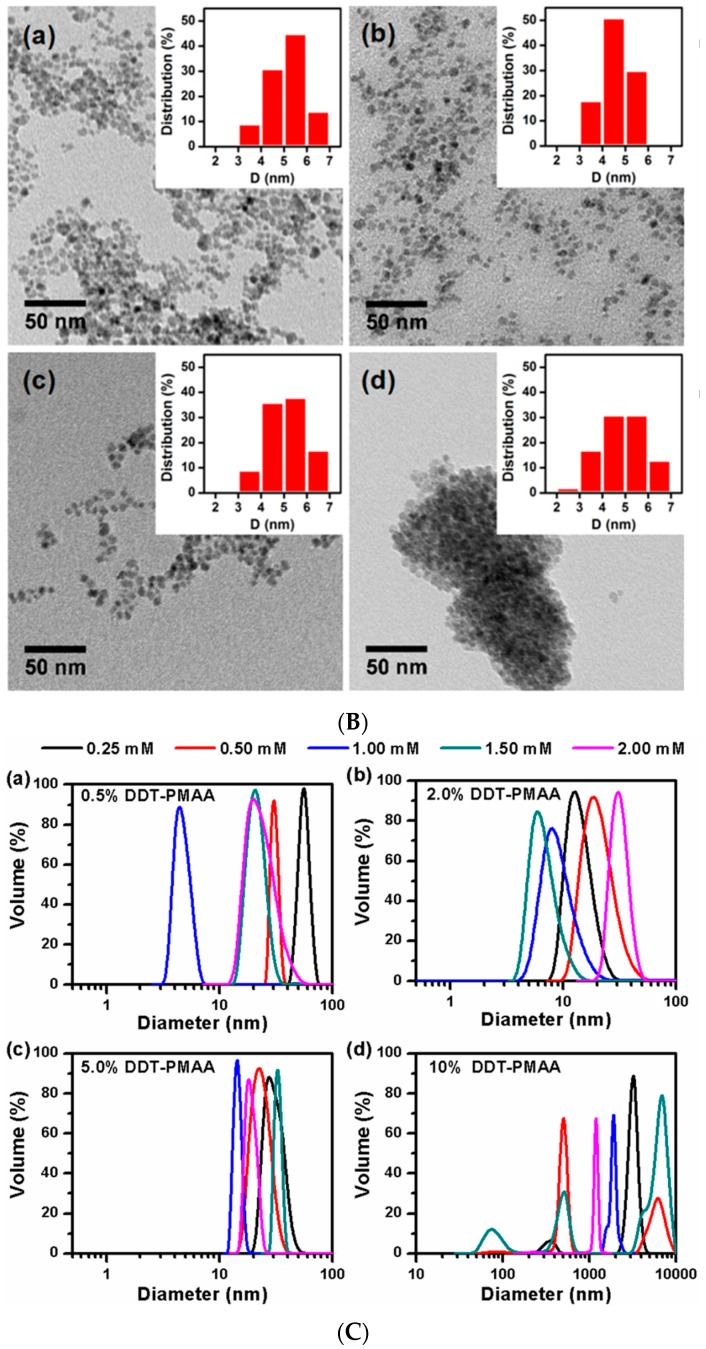
(**A**) Structural presentation of MIONs@DDT–PMAA. (**B**) and (**C**) TEM images and DLS curves along with particle size distribution histograms of MIONs prepared with (**a**) 1.5 mM of 0.5% DDT–PMAA (**b**); 2% DDT–PMAA (**c**); 5% DDT–PMAA (**d**) and 10% DDT–PMAA [7].

Encouraged from the previous work, Tan, *et al.* further explored and reported the MIONs preparation using a novel multifunctional, biocompatible and water-soluble thioether end functionalized polymer ligand dodecanethiol-polymethacrylic acid (DDT–PMAA) via a co-precipitation method. The ultra-small magnetic iron oxide nanoparticles (0.7–4.6 nm, MIONS) were fairly monodisperse and highly water soluble with high saturation magnetization (50 emu·g^−1^, Figure 6). DDT–PMAA efficiently controls the size of the MIONs and provides excellent water solubility, long time stability against aggregation, and oxidation. These MIONs are biocompatible and their multifunctional surface, rich in thioether and carboxylic acid groups, makes them attractive for many bio-applications [57].

## 6. Fluorescent Metal Nanoclusters

Recently, a new class of fluorescent metal nanoclusters has emerged and attracted enormous attention for its unique properties and applications [5,85,86]. These metal NCs are composed of a few to hundreds of atoms and possess several distinct optical, electrical, and physical/chemical properties compared to metal nanoparticles [87]. As these metal NCs are comprised of a few to hundreds of atoms this leads to a change of their continuous density of states to discrete energy levels. The metal NCs, in fact, bridge the gap between molecules and particles and exhibit the properties of both, simultaneously [85]. New synthetic protocols are extensively being explored for the synthesis of monodisperse metal NCs, because of their promising applications in the field of optoelectronics, bio-imaging, biological sensing, and catalysis. A commonly employed method is the reduction of metal precursors or etching of large nanoparticles in the presence of suitable reducing agents and a stabilizing scaffold such as small thiol molecules, polymers, and biomolecules. Furthermore, the nature of the scaffold is responsible not only to control their size but also to influence their fluorescence by the charge transfer from the surface bound ligands to the core metal atoms [88]. A breakthrough in preparing highly fluorescent Au nanoclusters was achieved by Zheng and co-workers [87,89,90]. The poly(amidoamine) (PAMAM) dendrimers were used as a stabilizer which itself imparts fluorescence and NaBH_4_ as a reducing agent. The complex coordination between Au^3+^ (Au^+^) ions and the amino or carboxylic groups of PAMAM generated a series of Au_5_, Au_8_, Au_13_, Au_23_, and Au_31_ nanoclusters with an intense blue florescence and 41%quantum efficiency. By varying the molar ratios of Au^3+^ and PAMAM from 1:1 to 1:15, the emission of these Au nanoclusters were tuned from the ultraviolet (UV) to the near infrared (NIR) range with a QY from 10% to 70%. Fluorescent Ag NCs were also prepared by following the same protocol. The major drawback of these NCs is the use of fluorescence dendrimers, which complicated the originality of the fluorescent property of the nanoclusters [91,92].

**Figure 7:**
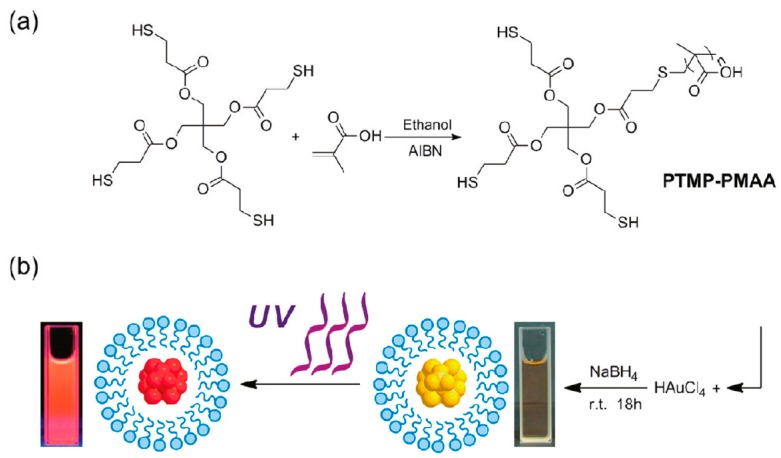
(**a**) Reaction scheme for the synthesis of thioether polymer ligand PTMP–PMAA; (**b**) Preparation of Au NCs stabilized by polymer ligands. Inserts are photographs of Au NCs’ aqueous solution under the irradiation of 365 nm ultraviolet light (**left, red** fluorescence) and day light (**right**, **yellow** color) [94].

Tan, *et al.* also reported the synthesis and applications of Au, Ag, and Cu NCs using polymers containing multiple thiol groups. The nanoclusters were smaller than 5 nm and fluoresced in the visible to near-infrared (NIR) region. Because of their long lifetime, large Stokes shift, and biocompatibility, these NCs represent an interesting material for sensing and imaging [93]. Tan, *et al.* further exploited the use of multidentate thioether-terminated poly-(methacrylic acid) (PTMP–PMAA) as capping ligand to produce metal nanoclusters (Figure 7). This polymer is comprised of a linear chain of water-soluble methacrylic acid units and a hydrophobic end group consisting of, on average, three unreacted thiols and one thioether functionality. Due to the strong steric effect, this polymer ligand can effectively control the size of NCs. Thus, by varying the polymer to gold precursor ratio, the size of the NPs can be tuned and the transition from non-fluorescent to fluorescent can be attained with the core diameter between 1.7 and 1.1 nm respectively. In contrast to dendrimers, the polymer ligands do not impart fluorescence, and the observed fluorescence is possibly because of the Au nanoclusters [56,94].

**Figure 8:**
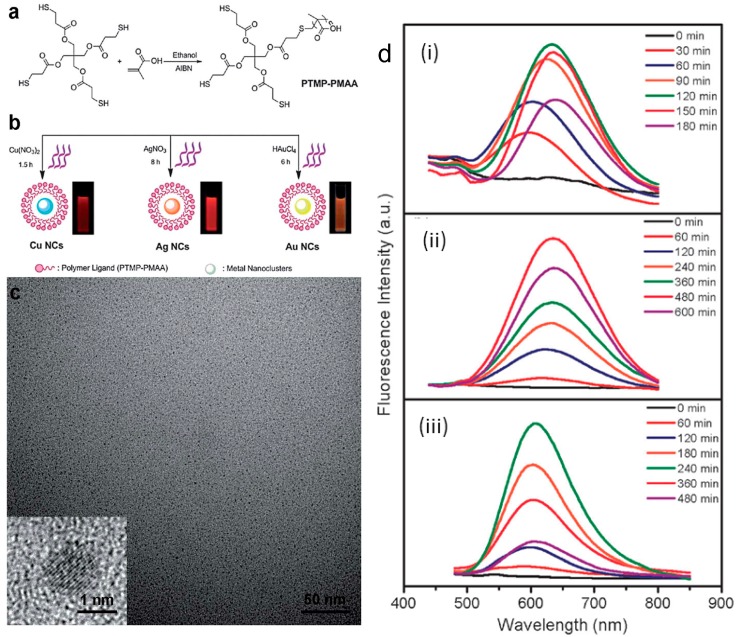
Schematic diagram of preparation of (**a**) the polymer ligand PTMP–PMAA; (**b**) photoreductive synthesis of fluorescent Cu, Ag, and Au nanoclusters; (**c**) TEM image of Au nanoclusters; and (**d**) Time dependent evolution of fluorescence emission spectra of the solution containing (**i**) PTMP–PMAA and Cu(NO_3_)_2_ (excited at 360 nm); (**ii**) PTMP–PMAA and AgNO_3_ (excited at 405 nm); (**iii**) PTMP–PMAA and HAuCl_4_ (excited at 360 nm) upon UV-irradiation at 365 nm [5].

Tan, *et al.* also reported the preparation of fluorescent Au, Ag, and Cu nanoclusters using photoreduction *i.e.*, a clean and non-toxic approach, in the presence of polymers (Figure 8). The quantum yield (QY) of the resultant Au, Ag, and Cu nanoclusters was 5.3%, 6.8%, and 2.2%, respectively [5]. Shang, *et al.* also prepared highly fluorescent Ag nanoclusters (18.6% QY) in the presence of poly(methacrylic acid) (PMAA) using the photoreduction approach [95]. Compared with PMAA, thioethers containing polymer ligands have a stronger steric hindrance effect. The strong luminescence emission is attributed to aggregation induced emission of Au(I)-thiolate complexes [5]. In order to further investigate the polymer hindrance effect, we also designed different derivatives of multidentate thioether terminated polymer ligands including, poly(methyl methacrylate) (PTMP–PMMA), poly(*n*-butyl methacrylate) (PTMP–PBMA), and poly(*t*-butyl methacrylate) (PTMP–PtBMA and successfully synthesized water soluble fluorescent Au NCs by the reduction of HAuCl_4_ with NaBH_4_. The Au NCs exhibit blue emission florescence due to their small particle size and decent quantum yield (QY) of 3.8%, 14.3%, and 20.1%, respectively, which increases with increasing polymer steric hindrance, *i.e.*, PTMP–PMMA < PTMP–PBMA < PTMP–PtBMA [88].

## 7. Applications of Nanoparticles/Clusters Capped with Thioether Based Ligands

The thioether ligands have the ability to tune and control the morphology of nano-structured inorganic and organic particles and the previous work on such types of materials has proven their long term stability in the biological as well as the chemical environment. Various applications of thioether capped nanoparticles have been explored in the past decade including those in biolabelling, sensing, drug delivery, MRI, and catalysis. Nanomaterials like silica NPs [96], quantum dots [97], carbon nanoclusters [98], fluorescent clusters of noble metals [94,99] have been vastly used in bioimaging and biolabelling. It is difficult to cover completely all such nanomaterials demonstrated for bioimaging and is not even within the scope of this review. We will try to focus only on the applications of metal nanoparticles/nanoclusters coated with thioether based ligands/polymers.

### 7.1. Bio-Imaging by MWPs Functionalized Nanoparticles

**Figure 9:**
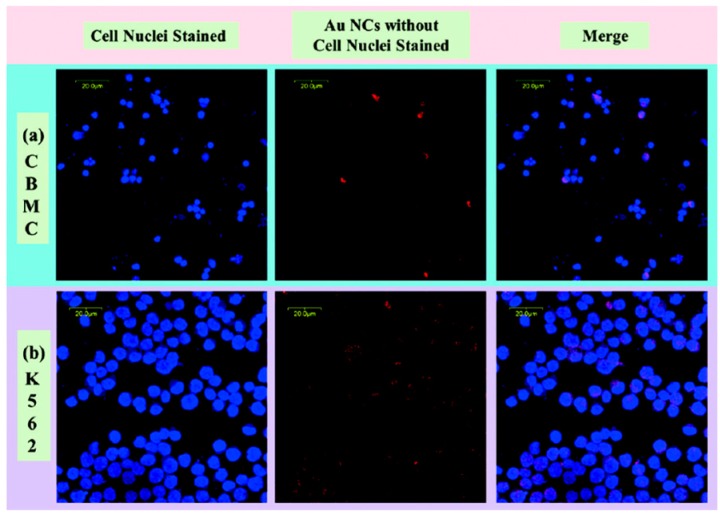
Confocal microscopic Images of (**a**) CBMC; normal cells; (**b**) K562; cancer cells, after incubation with Au NCs in a medium containing fetal calf serum (FCS) for 24 h. The nuclei of cells were tainted with Hochest-33258 to yield blue fluorescence and the red fluorescence is due to the presence of Au NCs [94]. The scale bar is 20 µm.

Fluorescent metal nanoclusters, especially of gold and silver, are considered to have more potential and are thus attractive materials for biosensing, optical imaging, and drug delivery, *etc.* It is because; they are fairly biocompatible, photo-stable, have tunable solubility in aqueous or organic media, have size dependent optical properties, have better contrast, and are fairly easy to characterize using various available analytical tools. For example, Huang and his coworkers prepared very stable Au NCs by using thioether based ligand (PTMP–PMAA). These NCs do not photo-bleach easily and can maintain fluorescence up to 70% on irradiation with monochromatic light of 480 nm for 200 min, whereas rhodamine 6 G can only maintain 10% fluorescence after equivalent exposure to radiation. The MTT assay of PTMP–PMAA capped Au NCs and mercaptopropionic acid capped fluorescent quantum dots of cadmium and tellurium (MPA–CdTe) was performed on normal (cord blood mononuclear cells, CBMC) and cancerous (K562) hematopoietic cell lines. The nuclei of both normal CBMC and cancerous K562 hematopoietic cells were destroyed on taking quantum dots of MPA–CdTe. The cellular activity of normal CBMC was also observed to decrease two folds by adding MPA–CdTe quantum dots than that with PTMP– PMAA capped Au NCs. It was finally concluded that Au NCs were better uptaken by hematopoietic cancer cells than normal cells and no acute toxicity was observed in comparison to quantum dots of MPA–CdTe (Figure 9) [94].

### 7.2. Detection of Metal Ions by MWPs Functionalized Nanoparticles

Fluorescent materials are also quite useful to sense organic and inorganic pollutants. It is because; fluorescent materials are highly sensitive, simple to prepare, and a diverse class of such materials is now available. Their selectivity can be tuned by controlling their surface chemistry, *i.e.*, by choosing an appropriate ligand and modifying the end functionalities at their surface. Au NCs capped by multidentate thioether ligand (PTMP–PMAA) were found to be much selective in the detection of Cu^2+^, Pb^2+^, and Ag^+^ ions. For this, nitrate and chloride (0.2 M) solutions of Cd^2+^, Al^3+^, Pb^2+^, Cu^2+^, Ag^+^, Au^3+^, Pt^4+^, Ni^2+^, Co^2+^, K^+^, Na^+^, Ca^2+^, Mn^2+^, Zn^2+^, and Mg^2+^ were selected to examine their effect on the fluorescence intensity of Au NCs. The salts of Cu^2+^, Pb^2+^, and Ag^+^ were found to quench fluorescence leading to the aggregation and precipitation of Au NCs. It is because, these metal ions formed chelate complexes with the PTMP–PMAA ligand. This may lead to their potential application for the detection of metal ions (Cu^2+^, Pb^2+^, and Ag^+^) in unrevealed biochemical/ chemical systems. Other than sensing, the Au NCs can also be prospective candidates in disease diagnostics, drug delivery, and as MRI contrast agent. This is because; they are photostable in aqueous media with excellent near-infrared emission, biocompatible with no detectable acute cellular toxicity, and very selective for cancerous cells [94].

### 7.3. Drug Delivery by MWPs Functionalized Nanoparticles

**Figure 10:**
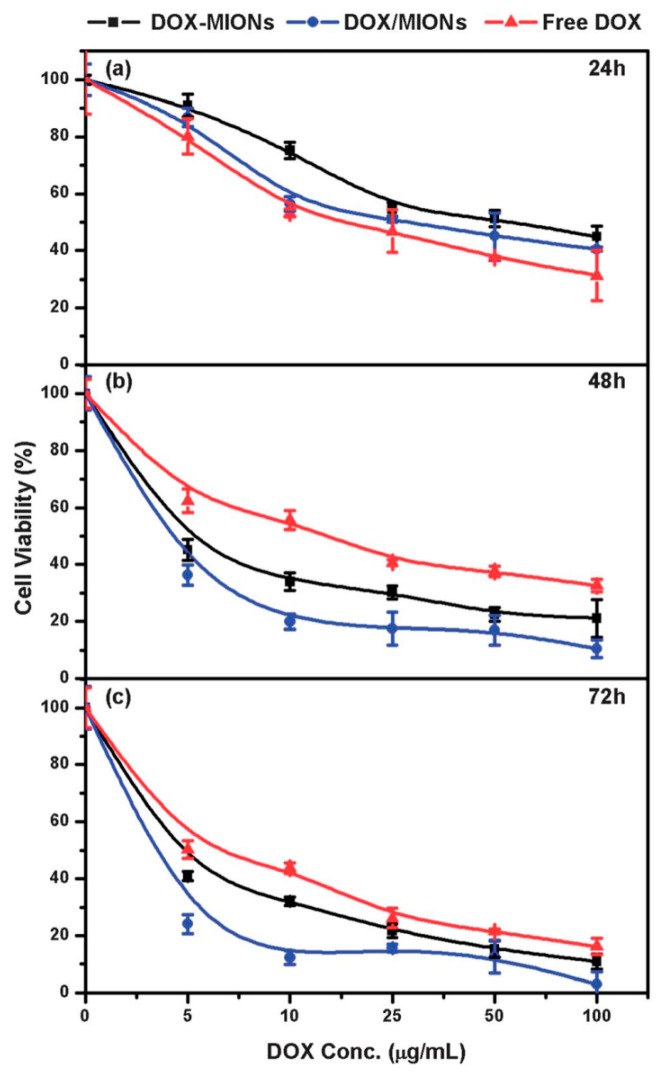
Viability of cells (HepG2) after (**a**) 24 h; (**b**) 48 h; and (**c**) 72 h incubation with DOX–MIONs, DOX/MIONs, and free DOX having equivalent concentration of DOX [7].

Thioether ligand capped nanoparticles have also been explored to determine their potential role in drug delivery. For example, Majeed, *et al.* reported MIONs capped with DDT–PMAA rich in carboxylic and thioether moieties, which were highly water soluble, stable, and monodisperse. The *in vitro* cytotoxicity assay revealed that polymer or polymer capped MIONs had no cellular toxicity up to 1000 µg/mL, whereas bare MIONs were found to show cytotoxicity above 25 µg/mL. The carboxylic groups of DDT–PMAA capped MIONs were further conjugated to amine groups of anticancer drug (doxorubicin, DOX) covalently and electrostatically to produce covalently linked DOX–MIONs and electrostatically linked DOX/MIONs respectively. The efficacy of DOX–MIONs, DOX/MIONs and free DOX were checked by incubating them with HepG2 for 24, 48, and 72 h [7]. It was observed that cell viability of HepG2 cells was decreased by increasing exposure of DOX in the cell medium, introduced either in free form or by its conjugate with MIONs. It was in accordance with previously reported results [100]. The cell viability of free DOX was reduced to 50% after 24 h and its anticancer activity was greater in comparison to its conjugates with MIONs. The cellular uptake of free DOX was by diffusion and greater than that of conjugated MIONs. This may be because of slower cellular uptake of conjugated DOX–MIONs and DOX/MIONs by endocytosis, which resulted in lesser anticancer activity after 24 h (Figure 10a) [100,101]. The DOX/MIONs and DOX–MIONs exhibited higher anticancer activity than free DOX in MTT assay for 48 and 72 h (Figure 8b,c). The higher activity of drug conjugates of MIONs was attributed to their greater cellular uptake at later stages and high solubility in cytoplasm due to the presence of thioether and carboxylic moieties. In conjugates of MIONs, the electrostatically linked DOX/MIONs demonstrated higher anticancer activity by fast releasing of DOX than covalently linked DOX–MIONs. This may be because DOX/MIONs release DOX into cytoplasm fairly easily as compared to DOX–MIONs, from where DOX penetrates into the nucleus and binds with DNA to arrest its functions to cause cell death [7,102]. Therefore the thioether based formulations of nanoparticles for drug delivery systems and sensing, may add an extra feature to work in the aqueous environment at physiological temperature and pH. Different hydrophilic and biocompatible polymers without any functionality to be ligated on metal nanoparticles are imparted with thioether bonds to use them as good protecting ligands and enhance hydration on the surface of nanoparticles [103,104], which is subsequently very important in defining the pharmacokinetics of drug loaded nanoparticles [105].

### 7.4. MWPs Functionalized Nanoparticles as MRI Contrast Agents

The examination of internal body parts is being improved by the use of MRI contrast agents in magnetic resonance imaging (MRI) [106]. The magnetic nanomaterials enhance their visibility by increasing the *R_2_* relaxivity, which is proportional to the surface area, concentration, and magnetization of metal NPs. A better contrast can be achieved by decreasing the size of metal NPs, and the nature of doping elements and other surface capping functionalities [107,108,109]. The movement and position of magnetic NPs, incorporated in the cell (*in vivo*) and body can be tracked by MRI for drug delivery and diagnostics related applications. In this regard, ultra-small super-paramagnetic nanoparticles (particularly iron oxide and cobalt) have been studied to explore their potential applications in MRI by many researchers [61,110,111]. For example, Jun, *et al.* observed an increase in *R_2_* value by increasing the size of the magnetic NPs. They used 2,3-dimercaptosuccinic acid and succeeded in preparing magnetic NPs having diameter of 4, 6, 9, and 12 nm with high *R_2_* value, *i.e.*, 78, 106, 130, and 218 mM^−1^·s^−1^ respectively under an applied field of 1.5 Tesla [112]. Recently, iron oxide nanorods capped with polyethyleneimine having chain length of 30, 40, 50, 60, and 70 nm with corresponding diameter of 4, 6, 9, 12, and 16 nm have also been reported. The magnetic field induced on these rods is found to be very high compared to previously reported spherical assemblies. These nanorods have high (*R_2_*) values, *i.e.*, 312, 381, and 608 mM^−1^·s^−1^ respectively and are proven to be good contrast agents in MRI at 3 Tesla [113]. Ian, *et al.* prepared two different polymers P1 and P2 (having *M*w 6800 and 6000 g·mole^−1^ respectively) of poly(*N*-isopropyl-*co*-*t*-butylacrylamide) containing thioether and terminal carboxylic functionalities. They used their –COOH moiety to stabilize respective cobalt and γ-Fe_2_O_3_ NPs of 7 ± 1 nm and 8 ± 1 nm respectively. The lowest critical solution temperature (LCST) of P1 and P2 was 23.5 and 28 °C. The NPs of cobalt and γ-Fe_2_O_3_ capped with P1 and P2 respectively were found to be thermoresponsive. The LCST of γ-Fe_2_O_3_ NPs was 37 °C in both phosphate buffer solution (PBS soln., pH 7.4) and water, whereas that of Co NPs was above 37 °C in PBS buffer and 25 °C in water. Therefore, cobalt NPs can also be used as MRI contrast agents for several biological systems. These cobalt NPs are stable and are not very harmful, as there is no significant toxicity reported for cobalt nanoparticles injected in living bodies. The cobalt NPs can, however, be toxic if oxidized to Co (II) and discharged in the body. This can only be avoided by preparing stable Co NPs [114]. Parkes and her colleagues prepared water soluble ultra-small cobalt NPs of 3.3 and 3.9 nm by using different concentrations and molecular weights. Both of these Co NPs were found to have higher (*R_2_*) relaxivity value 88 ± 32 mM^−1^·s^−1^ than that reported for superparamagnetic iron oxide NPs of the same size. Josephson and his colleagues also reported the use of near-infrared fluorescence (NIRF) materials in conjunction with MRI contrast agents for combined NIRF and MRI. They prepared cross-linked amine functionalized magnetic nanoparticles (amine-CLIO) by using aminated dextran and conjugated with argenyl peptides (R4) by disulphide (SS) and thioether (SC) linkages with subsequent attachment of indocyanine dye (Cy5.5) to yield Cy5.5-R4-SS-CLIO and Cy5.5-R4-SC-CLIO NPs probes. The fluorescence of Cy5.5 in both types of NPs probes was quenched. It was revealed from *in vitro* studies that the NIRF region of Cy5.5-R4-SS-CLIO NPs was restored on the addition of DDT, whereas that of Cy5.5-R4-SC-CLIO NPs was restored by trypsin. It is notable that the trypsin can only cleave the basic sequence of R4 and restore fluoresce of Cy5.5, when it is linked with the thioether bond in the corresponding probe. For this purpose, the Cy5.5-R4-SC-CLIO NPs probe (2.2 mg Fe/kg, 0.85 nmol dye, 30 μL) was injected subcutaneously to nu/nu nude mouse, and further joint MR/Optical imaging was utilized to give information about location and molecular environment of probe by MRI and NIRF contrast agents respectively [115].

### 7.5. Hyperthermic Tumor Therapy

Hyperthermia tumor therapy has traditionally been explored for the detection/treatment of tumor cells that are more heat-sensitive than normal cells [116]. Particularly iron oxide NPs are considered as a potential candidate for cancer hyperthermia therapy due to their well-known biocompatibility. Magnetic nanoparticles of smaller size exhibit a superparamagnetic property at a certain temperature range and are, therefore, potential candidates for hyperemia related applications [117]. Hyperthermia potential of magnetic nanoparticles is particularly influenced by their composition, shape, coating, magnetic properties, size, and size distribution. Recently some studies have been carried out to investigate the influence of these properties on the resulted hyperthermia treatment effectiveness. Kolhatkar, *et al.* reviewed some parameters that can be varied by switching the magnetic properties of NPs. Carrey, *et al.* described a method to identify the suitability of materials for hyperthermia [116]. Likewise, Chen, *et al.* reported magnetic microarray chips, by coating graphite on highly magnetic FeCo through a chemical vapor deposition method. These chips could easily capture and efficiently detect cancer cells from one mL of blood [118].

### 7.6. Nanoparticles as Catalysts (Selected Examples)

**Figure 11:**
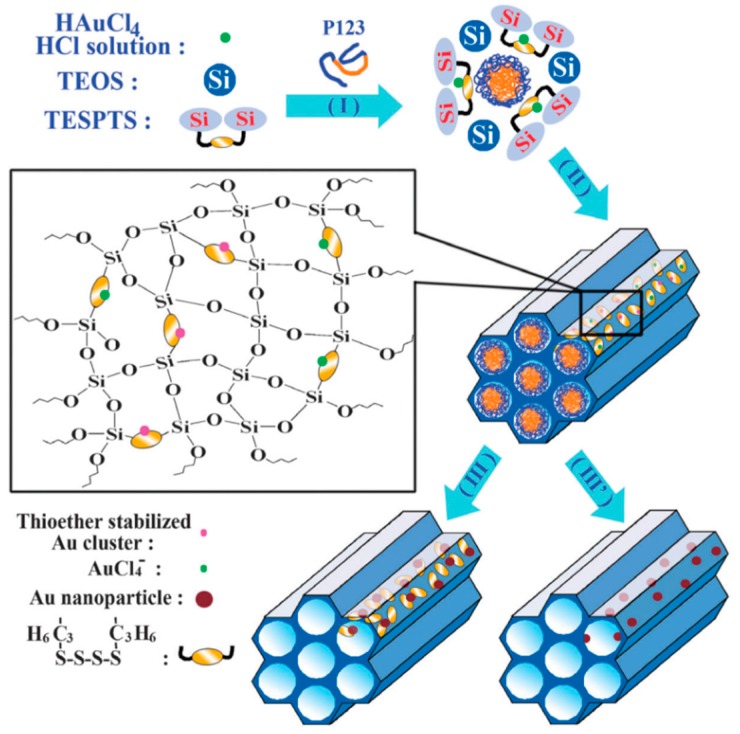
Formation of Au species during the one-pot synthesis process: (**I**) mixing of all reaction precursors in one pot; (**II**) hydrothermal treatment at 100 °C for 24 h; (**III**) ethanol extraction followed by H_2_ reduction; and (**III′**) calcined at high-temperature [133].

Cyclohexanol and cyclohexanone (K/A oil) are produced during the oxidation of cyclohexane. They are converted to adipic acid, which is one of the main precursors of Nylon and 6 × 10^6^ tons of it are produced per annum [119]. The main source for production of adipic acid is normally through oxidation of KA oil by HNO_3_. The K/A oil is produced by the oxidation of cyclohexane using homogenous catalysts using transition metal complexes; such as porphyrins of cobalt [120], chromium, and manganese [121,122], and others μ-oxo-bisiron(III)porphyrin [122]. The homogeneous catalysis exhibits certain limitations including the requirement of high temperature and pressure, low product yield in this case, and the difficulty in separation of product from catalyst at the end of the reaction [123]. These deficiencies can be overcome to some extent by the use of heterogeneous catalysts such as titanium based aluminophosphates and silicates [124,125] lantanides (La, Ce, Sm, Dy, Y, Gd) containing aluminophosphates [126], silver nanoparticle loaded mesoporous molecular sieves (Ag/MCM-41), There are, however, some shortcomings in this particular reaction, *i.e.*, poor yield and selectivity that remained a challenge. To address these shortcomings, nano-structured catalysts like nanoparticles of Fe_2_O_3_, Co_3_O_4_ or mixed oxides of Fe and Co were developed to improve the yield of K/A oils [127]. Noble metals have also been reported to enhance yield and selectivity, e.g., the composite of carbon quantum dots and gold nanoparticles in the size range of 5–15 nm have been found highly effective in converting 63.8% of cyclohexane to cyclohexanone with 99.9% selectivity and having 10 times the recyclability [128]. Moreover, gold nanoparticles are also proven to be good candidates for the production of K/A oil. For example, Au NPs capped with silica based ligands having thiol [129,130] chloro [129], and amine [130,131] functionalities were supported on mesoporous silica with a fine distribution of Au NPs. These catalysts showed good activity for the oxidation of cyclohexane and n-alkane respectively. Similarly, silica based thioether ligand (Bis(triethoxysilyl) propane tetrasulfide, TESPTS) was used by Pingping, *et al.* to prepare mesoporous silica (TESPTS–SiO_2_) loaded with Au complexes. It is because; the thioether groups of TESPTS provide a good complexation with AuCl_4_ complexes and lead to a good dispersion of Au on (TESPTS–SiO_2_). Later on, these were hydrothermally treated to prepare a framework of mesoporous silica immobilized with thioether stabilized Au NPs (Au/TESPTS–SiO_2_). The templating agents were removed by ethanol extraction followed by the reduction of the remaining (thioether–anionic Au) complexes with hydrogen to yield Au NPs 2–6 nm loaded silica of light brown colored (Au/TESPTS–SiO_2_–H_2_), followed by calcination at 500 °C to prepare ligand free silica (Au/TESPTS–SiO_2_–cal) loaded with ruby red Au NPs in the size range of 3–7 nm (Figure 11). The red color indicates the presence of nanoparticles [132]. Au NPs on Au/TESPTS–SiO_2_–cal were evenly dispersed and more exposed for chemical reaction than those in the Au/TESPTS–SiO_2_–H_2_ catalyst. This was because the calcined catalyst “Au/TESPTS–SiO_2_–cal” resulted in greater selectivity and efficiency in converting cyclohexane to K/A oil than Au/TESPTS–SiO_2_–H_2_ [133].

## 8. Conclusions

This review summarizes recent developments to control the morphology and surface properties of metal NPs/NCs protected by thioether end-functionalized ligands. The thioether end-functionalized polymers particularly have been found to be very useful to produce a variety of metal/metal oxide nanoparticles of uniform size. These polymers also can affect the surface chemistry and hydrophobicity/hydrophilicity of the nanoparticles by controlling the chemical composition and functional groups in their backbone. Other functional groups in such polymers, particularly carboxylic and amine groups, offer a simple and facile strategy to further control/modify the surface chemistry of these nanoparticles. Due to the weaker thioether-metal interaction compared to that in the thiol-metal covalent bond, thioether ligands can easily be replaced with a variety of other ligands, especially those containing a thiol (–SH) group including biomolecules and alkanethiols and facilitate nanoparticles phase transfer from aqueous to organic or media and/or *vice versa*. The easier control over the size, surface chemistry, and solubility of such stable metal/metal oxide nanoparticles renders them attractive candidates for potential applications in bio-imaging, sensing, drug delivery, MRI contrast agents, and catalysis.

## Abbreviations

The following abbreviations are used in this manuscript:

MNPs	Metal nanoparticles
MNCs	Metal nanoclusters
MRI	Magnetic resonance imaging
MIONs	Magnetic iron oxide nanoparticles
MFH	Magnetic fluid hyperthemia
MWPs	Multifunctional water soluble polymer ligands
PEG	Polyethylene glycol
SAM	Self assembled monolayers
DDT	Dodecanethiol
PVAc	Polyvinyl acetate
PMAA	Polymethyl acrylic acid
PVP	Polyvinyl pyrolidone
PVA	Polyvinylacetate
PEI	Polyethyleneimine
PAMAM	poly(amidoamine) dendrimers
DMAP	4-(dimethylamino)pyridine
CTAB	Cetyl trimethylammonium bromide
MNPs	Magnetic nanoparticles
TOAB	Tetraoctyl ammonium bromide
PtBMP	poly(*t*-butyl methacrylate)
PBMA	poly(*n*-butyl methacrylate)
DOX	Doxorubicin
PBS	Phosphate buffer saline
CD	Cyclodextrin
DLS	Dynamic light scattering
